# Comparison of Biofilm Formation between Major Clonal Lineages of Methicillin Resistant *Staphylococcus aureus*

**DOI:** 10.1371/journal.pone.0104561

**Published:** 2014-08-08

**Authors:** Evelyn Vanhommerig, Pieter Moons, Daniel Pirici, Christine Lammens, Jean-Pierre Hernalsteens, Henri De Greve, Samir Kumar-Singh, Herman Goossens, Surbhi Malhotra-Kumar

**Affiliations:** 1 Department of Medical Microbiology, University of Antwerp, Antwerp, Belgium; 2 Vaccine & Infectious Disease Institute, University of Antwerp, Antwerp, Belgium; 3 Molecular pathology Group, Cell Biology and Histology, University of Antwerp, Antwerp, Belgium; 4 Department of Research Methodology, University of Medicine and Pharmacy of Craiova, Craiova, Romania; 5 Viral Genetics Research Group, Vrije Universiteit Brussel, Brussels, Belgium; 6 Structural Biology Brussels, Vrije Universiteit Brussel, Brussels, Belgium; 7 Structural and Molecular Microbiology, Structural Biology Research Center, VIB, Brussels, Belgium; Columbia University, United States of America

## Abstract

**Objectives:**

Epidemic methicillin-resistant *S. aureus* (MRSA) clones cause infections in both hospital and community settings. As a biofilm phenotype further facilitates evasion of the host immune system and antibiotics, we compared the biofilm-forming capacities of various MRSA clones.

**Methods:**

Seventy-six MRSA classified into 13 clones (USA300, EMRSA-15, Hungarian/Brazilian etc.), and isolated from infections or from carriers were studied for biofilm formation under static and dynamic conditions. Static biofilms in microtitre plates were quantified colorimetrically. Dynamic biofilms (Bioflux 200, Fluxion, USA) were studied by confocal laser-scanning and time-lapse microscopy, and the total volume occupied by live/dead bacteria quantified by Volocity 5.4.1 (Improvision, UK).

**Results:**

MRSA harbouring SCC*mec* IV produced significantly more biomass under static conditions than SCC*mec* I–III (P = 0.003), and those harbouring SCC*mec* II significantly less than those harbouring SCC*mec* I or III (P<0.001). In the dynamic model, SCC*mec* I–III harbouring MRSA were significantly better biofilm formers than SCC*mec* IV (P = 0.036). Only 16 strains successfully formed biofilms under both conditions, of which 13 harboured SCC*mec* IV and included all tested USA300 strains (n = 3). However, USA300 demonstrated remarkably lower percentages of cell-occupied space (6.6%) compared to the other clones (EMRSA-15 = 19.0%) under dynamic conditions. Time-lapse microscopy of dynamic biofilms demonstrated that USA300 formed long viscoelastic tethers that stretched far from the point of attachment, while EMRSA-15 consisted of micro-colonies attached densely to the surface.

**Conclusions:**

MRSA harbouring SCC*mec* types IV and I–III demonstrate distinct biofilm forming capacities, possibly owing to their adaptation to the community and hospital settings, respectively. USA300 demonstrated abundant biofilm formation under both conditions, which probably confers a competitive advantage, contributing to its remarkable success as a pathogen.

## Introduction

*Staphylococcus aureus* is a commensal that colonizes the nasal cavity of approximately 30% of the human population. However, in susceptible people or those predisposed with risk factors, this organism easily leads to infections, ranging from relatively mild skin and soft tissue infections to life-threatening infections such as sepsis, endocarditis and necrotising pneumonia. Although *S. aureus* infections used to be easily treatable with penicillins, its adaptability resulted in the rise of more successful clones and the discovery of penicillin resistance in the 1940s [1], and of methicillin-resistant *S. aureus* (MRSA) in the 1960s [2]. Methicillin resistance was due to the uptake of the staphylococcal cassette chromosome *mec* (SCC*mec*) encoding an alternative penicillin binding protein PBP2a with reduced affinity for beta-lactams [3]. The vast use of antibiotics in the hospital setting resulted in the acquisition of additional, often horizontally transmitted, genetic determinants that led to highly resistant hospital-acquired (HA) MRSA, sometimes harbouring more than 20 antibiotic resistance determinants [4]. Although these strains were highly fit for the hospital setting, they often had slower growth rates and a lower overall fitness compared to their community-associated counterparts. The community-acquired (CA-) MRSA arising in the 1990s [5] were therefore associated with the smaller SCC*mec* type IV-V elements, which resulted in the loss of the antibiotic resistance markers associated with the larger SCC*mec* type I–III elements while simultaneously leading to strains that were more fit to compete outside the hospital setting [6]–[7]. Recently however, the borders between CA- and HA-MRSA have started to fade with CA-MRSA invading the hospital environment.

MRSA are not only subdivided into HA- and CA-MRSA, but also into different clones, often originally being described based on geographical site of first isolation [8]. Clones can be genetically characterized based on the sequence type (ST) and clonal complex (CC) by multi locus sequence typing (MLST), by SCC*mec* typing or by the presence of certain virulence genes such as those coding for Panton Valentine leukocidin (*pvl*) or the Arginine Catabolic Mobile Element (ACME; *arc* and *opp* clusters) [9].

The ability of *S. aureus* to form biofilms is an important characteristic which complicates infections due to MRSA, especially those associated with foreign materials such as catheters and implants [10]. In biofilms, the surface-associated bacteria are encased in an extracellular matrix and are thus far more resistant to antibiotics, often resulting in the need to remove the infected device in order to be able to treat the infection [11]. Biofilm formation is therefore an important survival strategy employed by bacteria that facilitates a prolonged persistence of infection and increases human morbidity and mortality.

The aim of the present study was to compare the biofilm-forming capabilities of MRSA clones that were most commonly associated with infections worldwide. We hypothesized that biofilm formation might be more prevalent in the more virulent and non-multi-drug resistant (CA) MRSA harbouring SCC*mec* type IV, aiding their rapid spread and the ability to cause severe infections in the community.

## Materials and Methods

### Strain characteristics

Seventy-six well-characterized MRSA belonging to the 13 most important epidemic clones, based on MLST and SCC*mec* typing results, were selected for this study (Table 1). Of the 76 strains, 48 had been isolated from clinical infections (wounds/abscesses, n = 27; blood, n = 5; urinary tract, n = 3; and respiratory tract, n = 13), 1 from a stool sample, 15 from nares of healthy carriers and the rest were of unknown origin. MLST and SCC*mec* typing were performed as described previously [12]–[13]. Genetic relatedness was further confirmed by PFGE typing using *Sma* I [14]. Presence of *pvl* and the ACME I element (*arcA* and *opp3AB* gene clusters) were investigated by real-time PCR and PCR-sequencing, respectively [13], [15]. Antibiotic susceptibility testing by disc diffusion was carried out utilizing a panel of antibiotics: chloramphenicol, erythromycin, clindamycin, trimethoprim-sulfamethoxazole, rifampicin, gentamicin, ciprofloxacin, tetracycline and cefoxitin (Table S1).

**Table 1:** Overview of MRSA clone characteristics, site of isolation, and biofilm formation in static and shear flow assays.

MRSA clone (No. of strains)	Clonal complex	*SCCmec* type	Sequence type	Presence of *pvl/ACME*	Site of isolation	Biofilm formation in static assay**			Biofilm formation in shear flow assay	
						Strong formers (n)	Moderate formers (n)	Weak formers (n)	+ (n)	− (n)
Southern Germany (6)	5	I	228/5	−	Nares (n = 4) Respiratory tract (n = 1) Nk *(n = 1)	1	2	3	3	3
New York/Japan (4)	5	II	5/496	−	Abscess/wound (n = 2) Blood (n = 1) Nk (n = 1)	0	4	0	3	1
Iberian (4)	8	I	247/336	−	Abscess/wound (n = 1) Respiratory tract (n = 1) Stool (n = 1) Nk (n = 1)	1	2	1	2	2
Hungarian/Brazilian (12)	8	III	239/241	−	Abscess/wound (n = 5) Respiratory tract (n = 4) Nares (n = 1) Blood (n = 2)	5	3	4	8	4
EMRSA-16 (4)	30	II	36	−	Nares (n = 2) Abscess/wound (n = 1) Nk (n = 1)	1	2	1	3	1
USA600 (3)	45	II	45	−	Nares (n = 1) Nk (n = 2)	0	0	3	3	0
Pediatric (5)	5	IV	5	−	Abscess/wound (n = 3) Respiratory tract (n = 1) Nares (n = 1)	3	1	1	0	5
USA500 (8)	8	IV	8	−	Abscess/wound (n = 2) Nares (n = 2) Urinary tract (n = 1) Nk (n = 3)	8	0	0	3	5
USA300 (3)	8	IV	8	*pvl/ACME*	Abscess/wound (n = 2) Blood (n = 1)	3	0	0	3	0
EMRSA-15 (7)	22	IV	22	−	Nares (n = 2) Respiratory tract (n = 1) Blood (n = 1) Nk (n = 3)	7	0	0	5	2
South-West Pacific (2)	30	IV	30	−	Abscess/wound (n = 1) Respiratory tract (n = 1)	0	1	1	0	2
South-West Pacific (3)	30	IV	30	*pvl*	Abscess/wound (n = 3)	0	1	2	0	3
Berlin (11)	45	IV	45	−	Abscess/wound (n = 4) Respiratory tract (n = 4) Urinary tract (n = 2) Nares (n = 1)	3	6	2	5	6
European (4)	80	IV	80	*pvl*	Abscess/wound (n = 3) Nares (n = 1)	3	1	0	1	3

*Nk, Not known; **Defined cut-offs for strong, moderate, and weak biofilm formers (OD_492_: >0.027, 0.027–0.009, and <0.009, respectively).

### Biofilm formation in a static model

All strains were tested in triplicate on a 96-well microtitre plate with a polystyrene peg lid as described previously [16], with few modifications. Inoculum was prepared by diluting log phase cultures grown in brain heart infusion (BHI) broth enriched with 1% glucose to OD_600_ 0.04 after which 200 µl was dispensed in each microtitre plate well (n = 9/strain). Subsequently, microtitre plates were closed with peg lids and incubated at 37°C in air for 72 hours with medium replacement every 24 hours. A known biofilm-forming strain (*S. aureus* ATCC25923) and a strain consistently negative for biofilm formation (*S. aureus* 5374) were utilized as controls during each assay. After growth, the pegs were washed 3 times by submersion in PBS at room temperature, fixed with methanol and stained with Hucker's crystal violet (2%) for 15 minutes. Afterwards, pegs were rinsed with water and the stain eluted by submersion for 30 minutes in 200 µl of 33% acetic acid. OD values were measured at 492 nm (Multiskan FC photometer, Thermo Scientific), normalized to the blank and compared to 72 h-old biofilms of the positive control strain. Strains were divided into three groups based on previously published criteria: those showing on average (of three OD values) <25% biomass of the positive control strain were designated as weak biofilm formers, 25%–75% biomass as moderate biofilm formers, and ≥75% biomass of the positive control as strong biofilm formers [16].

### Biofilm formation, viability determination and quantification in a shear flow model

All strains were tested on a medium-throughput continuous flow system (BioFlux 200, Fluxion Biosciences, USA). The BioFlux 200 consists of a 48-well plate with a microchannel connection between 24 paired sets of input and output wells [17]. Electropneumatic regulators allow precise and individual control over the flow (and shear) rate applied in each channel. The same MRSA strain inoculum utilized in the static peg plate assay was also used to inoculate the BioFlux system by reversing the flow and pushing the bacteria into the microchannel from the output well, thus avoiding contamination of the input well. Bacteria were allowed to attach for one hour, followed by 17 hours of incubation at 37°C in BHI with a pressure of 0.5 dyne/cm^2^. Biomass in the microfluidic channels was visualised by addition of 2.5 µl of live/dead BacLight viability stain (Invitrogen, Life Technologies) to each of the input wells, followed by flowing the stain for 10 minutes at 0.5 dyne/m2 in the dark. Prior to staining, the microchannels were flushed with 100 µl of 0.85% NaCl at 0.5 dyne/cm2 for 10 minutes to wash away unattached or loosely bound bacteria. Biofilms were visualized with an inverted fluorescence microscope (EVOSfl, AMG). Confocal images were obtained with a microlens-enhanced dual spinning disk confocal microscope system (Ultra*VIEW*; PerkinElmer, Seer Green, UK) equipped with an argon-krypton laser source with two excitation lines (488 and 568) for excitation of FITC- and Cy3-like labels, respectively. Images were processed using Volocity 5.4.1 (Improvision, PerkinElmer, Seer Green, UK) and Imaris 7.3.1 software (Bitplane, Switzerland). Time-lapse microscopy of fully grown biofilms (17 hours of growth) under shear flow was performed using a high-definition digital camera (HDR-TG7VE, Sony, Japan). To quantify the total volume occupied by live or dead cells, biofilm stacks obtained by confocal laser microscopy (CLSM) were exported from Volocity followed by a 10-iterations blind deconvolution protocol on Sharp Stack (Image ProPlus AMS 7, Media Cubernetics). After deconvolution, contrasts for green and red cells were equally enhanced in all stacks after which volumes of red and green cells were measured using Volocity, applying automatic threshold settings.

### Statistical analysis

Statistical analysis of biomass formation in the static assay was performed using the R Project software (version 2.11.1). The influence of clonal complex, SCC*mec* type, site of infection, and presence of *pvl* and ACME (*arcA* and *opp3AB*) on biofilm formation and the comparison of the space occupied by the cells within the dynamic biofilms were analysed using one-way ANOVA, Welch's t-test for two-group comparisons using Bonferroni's post-hoc adjustments where indicated or a Pearson's χ^2^test.

## Results

### Biofilm formation in a static model

Cut-off OD values for weak (OD_492_<0.010), moderate (0.010–0.030), and strong (> 0.030) biofilm formers were defined as the 25% and 75% ratios of the average OD obtained for the positive control strain *S. aureus* ATCC25923 (OD_492_: 0.099, SD: 0.019) after correction for the blank (OD_492_: 0.058, SD: 0.008) [16]. Of the 76 strains tested in the static assay, 35 (46.1%) showed strong biofilm formation (average OD range: 0.034–0.393).

Significant differences in biofilm formation were observed between the various MRSA clones and SCC*mec* types (ANOVA, *F* = 2.28, df = 12, P = 0.018 and *F* = 2.83, df = 3, P = 0.044, respectively), with MRSA harbouring SCC*mec* IV producing significantly more biomass in the static biofilm model than those harbouring SCC*mec* I, II or III (P = 0.003, Bonferroni's adjusted α level 0.0125; 0.05/4), and those harbouring SCC*mec* II significantly less than those harbouring SCC*mec* I, III or IV (P<0.001). Twenty-five out of 41 (61%) strains harbouring SCC*mec* type IV were strong biofilm formers as opposed to 4 out of 11 (36%) strains harbouring SCC*mec* III, 2 out of 11 (20%) harbouring SCC*mec* I, and only 1 out of 11 (9%) harbouring SCC*mec* II.

**Figure 1:**
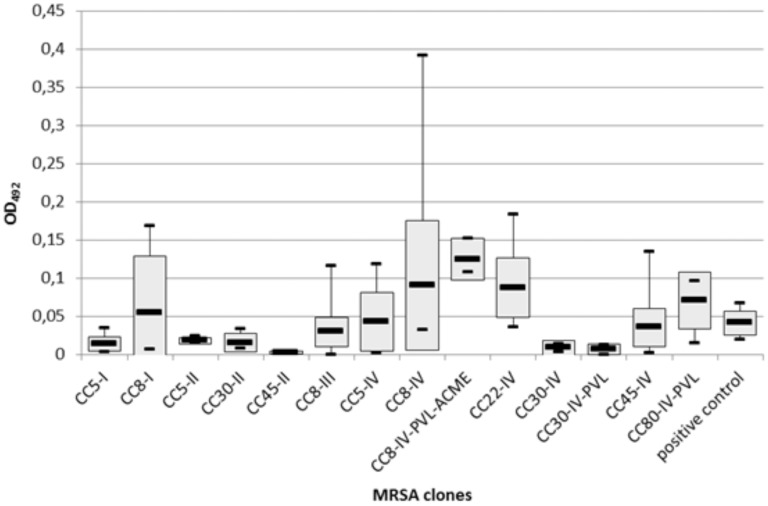
Box-whisker plot of biofilm formation by MRSA clones in the static assay. Boxes depict the 95% CI, black horizontal lines the average OD492 value, and the whiskers the OD492 value range (lowest and highest values) for each clone.

Within the different clones of the SCC*mec* IV group, all SCC*mec* IV strains belonging to CC22 (EMRSA-15, n = 7) and CC8 (USA300, n = 3; USA500, n = 8) exhibited strong biofilm formation with ODs ranging from 0.036–0.183 and 0.032–0.392, respectively (Figure 1), and average OD values of USA300 (OD_492_ = 0.125, SD = 0.024), USA500 (OD_492_ = 0.090, SD = 0.122), and EMRSA-15 (OD_492_ = 0.087, SD = 0.052) being the highest of the MRSA clones tested here. On the other end of the spectrum, the lowest average OD values were associated with the Southern German (OD_492_ = 0.014, SD = 0.012), New York/Japan (OD_492_ = 0.018, SD = 0.004), E-MRSA 16 (OD_492_ = 0.015, SD = 0.013), South-West Pacific (OD_492_ = 0.007, SD = 0.006) and especially, USA600 (OD_492_ = 0.002, SD = 0.001) clones. No correlation was observed between site of infection (P = 0.919) or presence of *pvl* (P = 0.187) and biofilm formation. In our study, the presence of ACME was uniquely associated with USA300 and thus high amounts of biomass (P = 0.011).

### Biofilm formation in a shear flow model

**Figure 2:**
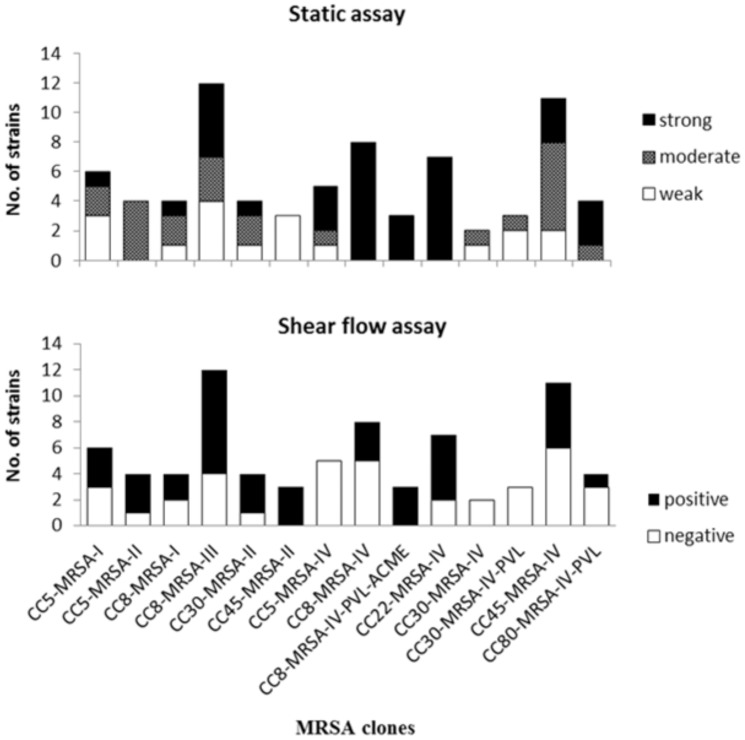
Performance of MRSA clones in the static and shear flow assay. A) In the static assay: biomass production was subdivided into weak, moderate and strong. B) In the shear flow assay biofilm formation was assessed as positive or negative.

**Figure 3:**
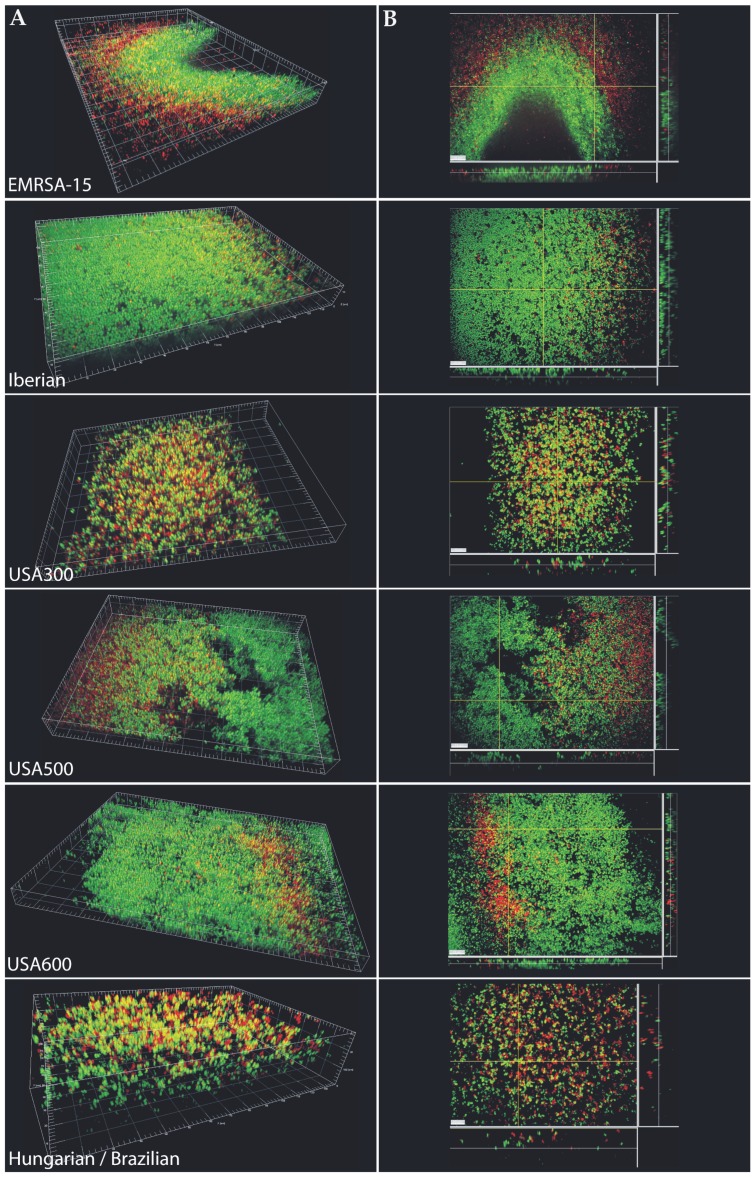
Live/dead BacLight staining and CLSM on 72-hour old MRSA biofilms under shear flow (Bioflux system). A) 3-dimensional representations. B) 2-dimensional projections, thin white lines in the xy plane depict the location of the section plane on the z axis. White bars have a length of 10 µm.

**Figure 4:**
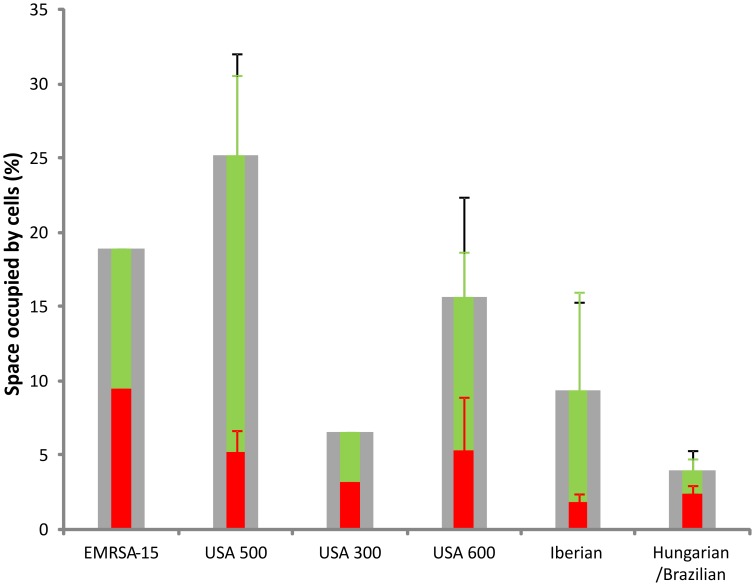
Space occupied by live (green) or dead (red) or total (grey) cells by different epidemic MRSA clones in a biofilm. Error bars denote standard deviations were calculated using 1 (USA300, EMRSA-15), 2 (USA600, Hungarian/Brazilian, Iberian) or 3 (USA500) independent z-stacks.

Of the 76 strains tested here, 39 (51%) strains successfully formed biofilms under shear flow. Successful biofilm formation was defined as the ability of cells to adhere to the surface in 2 of 2 or 3 independent experiments and was considered negative when no adherent cells could be shown in 2 of 2 or in 3 of 4 such experiments. Of the strains that were found positive for biofilm formation, 22 (66%) harboured SCC*mec* types I-III (n = 33) and 17 (40%) SCC*mec* type IV (n = 43) (P = 0.036, χ^2^). All USA600 (n = 3, CC45-SCC*mec* II) and USA300 (n = 3, CC8-SCC*mec* IV), but none of the South-West Pacific (n = 5, CC30-SCC*mec* IV) and Paediatric (n = 5, CC5-SCC*mec* IV) clones formed biofilms in the shear flow assay (Figure 2). The remaining 33 strains positive for biofilm formation under shear flow belonged to the following clones: Hungarian/Brazilian (8/12), EMRSA-15 (5/7), Berlin (5/11), EMRSA-16 (3/4), New York/Japan (3/4), Southern Germany (3/6), USA500 (3/8), Iberian (2/4) and European (1/4). On confocal microscopy, differences in the biofilms between various clones were evident, with the Iberian and USA600 clones forming thicker, more confluent and densely packed biofilms in comparison to those formed by USA500, USA300 and the Hungarian/Brazilian clones that showed gaps making the biofilms less confluent (USA500) or formed a less densely packed biomass (USA300 and Hungarian/Brazilian) (Figure 3). Since live/dead staining only stains bacterial cells and not the matrix, differences in cell densities within the biofilm could be captured through 3-dimensional quantification of the space occupied by micro-organisms. Both the USA300 and Hungarian/Brazilian clones demonstrated remarkably lower percentages of cell-occupied space (6.6% and 4.0%, respectively) compared to the other clones (EMRSA-15 = 19.0%, USA500 = 25.2%, USA600 = 15.7% and Iberian  = 9.4%) (Figure 3 and 4). Time-lapse microscopy demonstrated the strength of an MRSA biofilm under shear flow conditions (movie S1 and movie S2). Even when flow was 4× higher than normal growth conditions (2.0 dyne/cm^2^), the USA300 biofilm remained attached to the surface. Long elastic matrix strands kept the cells together. The USA300 (CC8-MRSA-IV-PVL-ACME) strain showed individual cells attached more to each other than to the surface resulting in the formation of long streamers/tethers, while attachment to the surface was more evident for the EMRSA-15 (CC22-MRSA-IV) strain (movie S1 and movie S2).

### MRSA showing consistent biofilm formation in static and shear flow assays

Sixteen of the 76 MRSA tested here consistently formed biofilms in both the static and shear flow assays; 19 strains were strong biofilm formers in the static assay and negative in the shear flow assay; 23 strains were weak or moderate biofilm formers in the static assay but positive in the flow assay; and 18 strains were negative in both assays. Of the 16 consistent biofilm formers in both the static and shear flow assays, 13 harboured SCC*mec* type IV and included all USA300 (n = 3), 5/7 E-MRSA15, 3/8 USA500, and 2/11 Berlin strains. Three MRSA harbouring SCC*mec* type III were also consistent biofilm formers in both assays and all belonged to the CC8-Hungarian/Brazilian clone.

## Discussion

### Influence of SCC*mec* on biofilm formation

Strains carrying SCC*mec* type IV were shown to have a significantly higher probability to form biofilms in the static assay compared to those with SCC*mec* type I–III, with strains carrying SCC*mec* type II being the least capable of biofilm formation. Conversely, in a shear flow assay strains harbouring SCC*mec* I–III showed higher potential for biofilm formation compared to strains carrying SCC*mec* IV.

Genetic differences in the SCC*mec* element harboured by HA- and CA-MRSA strains might underlie differential biofilm formation in static or dynamic conditions and are possibly related to the distinct requirements needed to accommodate community and hospital settings (e.g. skin and soft tissue infections versus catheter related or blood stream infections). For instance, phenol-soluble modulins (PSMs) are toxins that regulate virulence, and biofilm formation and structuring in *S. aureus* [18]. While most are chromosomally-encoded, *psm-mec* is present on the SCC*mec* II and III elements but not on SCC*mec* IV. *Psm-mec* has been shown to regulate the core genome-encoded PSMs, resulting in decreased virulence [19], and thicker more compact biofilms [20]. Interestingly, and also validating our *in vitro* results, MRSA harbouring SCC*mec* II and an intact *psm*-*mec* were found to be more frequently associated with catheter-related blood stream infections than strains with mutated *psm*-*mec* that showed PSM levels similar to SCC*mec* IV harbouring MRSA, and were also more likely to cause pneumonia and abscess formation [21].

Furthermore, differences in biofilm formation in the two models might be due to the different adherent surfaces utilized in the static (polystyrene) and dynamic (glass) systems. Surface-dependent attachment is especially more pronounced in *in vitro* setups where no human matrix proteins such as fibrinogen or fibronectin, which normally serve as an anchor for attachment on medical implants, are present [22].

### SCC*mec* IV harbouring EMRSA-15 and USA300 are prolific biofilm formers

In comparison to the other globally predominant MRSA clonal lineages, both USA300 and EMRSA-15 formed abundant biofilms. USA300 biofilms were, however, more structured compared to EMRSA-15 that were too dense to allow dye penetration into the deeper biofilm layers. Time-lapse microscopy showed that USA300 formed long viscoelastic streamers or tethers that stretched far from the point of attachment under shear flow, occasionally releasing parts of the biofilm. EMRSA-15 biofilms on the other hand consisted more of micro-colonies densely attached to the surface.

USA300 demonstrated to be an excellent biofilm former in both tested biofilm models, showing differences in biofilm characteristics compared to its USA500 progenitor and making its capacity to form biofilms an important characteristic to its transmission and survival, possibly contributing to its highly epidemic behaviour worldwide. The 31-kb genomic island ACME uniquely harboured by USA300 probably underlies its higher propensity to form biofilms. Recent data shows that the ACME-Arc system drives excessive production of host polyamines, which are uniquely toxic to *S. aureus*. However, the ACME also encodes a polyamine-resistance enzyme, SpeG, which combats excess host polyamines and enhances adherence to fibrinogen/fibronectin, resistance to antibiotic and keratinocyte-mediated killing and importantly, an enhanced biofilm forming capacity [23].

Thus, both the SCC*mec*IV and ACME elements seem to be major contributors to the extraordinary pathogenetic success of the USA300 clone by enhancing its virulence and biofilm forming capacities.

## Supporting Information

Table S1 **Antibiotic susceptibility data of MRSA clones.**
(DOCX)

Movie S1 **Time-lapse imaging of an EMRSA-15 biofilm under shear flow stress.** Shear stress was modulated as follows: time  = 0 s, pressure  = 0.5 dyne/cm^2^; t = 10 s, pressure  = 0 dyne/cm^2^; t = 30 s, pressure  = 0.5 dyne/cm^2^; t = 40 s, end.
(MTS)

Movie S2 **Time-lapse imaging of a USA300 biofilm under increasing shear flow stress.** Shear stress was modulated as follows: t = 0 s, pressure  = 0 dyne/cm^2^, t = 5 s, pressure  = 0.1 dyne/cm^2^, t = 10 s, pressure  = 0.2 dyne/cm^2^, t = 15 s, pressure  = 0.3 dyne/cm^2^, and so on till pressure  = 2.0 dyne/cm^2^ in the final 10 s of imaging. When the pressure is switched off at the end of the movie the long stretching tethers are most evident.
(MTS)
