# Supplementary material for: Comparison of Biofilm Formation between Major Clonal Lineages of Methicillin Resistant Staphylococcus aureus
Source: PLoS One. 2014 Aug 8;9(8):e104561. doi: 10.1371/journal.pone.0104561 (PMC4126748; doi:10.1371/journal.pone.0104561)
Supplement: Table S1 — Antibiotic susceptibility data of MRSA clones. (DOCX) [file pone.0104561.s001.docx]

| MRSA clone | Strain N° | chloramphenicol | erythromycin | clindamycin | trimethoprim+sulfamethoxazole | ciprofloxacin | tetracyclin | rifampicin | gentamicin |
| --- | --- | --- | --- | --- | --- | --- | --- | --- | --- |
| E-MRSA 15 | 1 | S | R | S | S | R | S | S | S |
|  | 2 | S | S | S | S | S | S | S | I |
|  | 3 | S | R | S | S | R | S | S | I |
|  | 4 | S | R | S | S | R | S | S | S |
|  | 5 | S | S | S | S | S | R | S | S |
|  | 6 | S | S | S | S | R | S | S | I |
|  | 7 | S | S | S | S | R | S | S | S |
| E-MRSA 16 | 1 | S | R | R | S | R | S | S | S |
|  | 2 | S | R | R | S | R | S | S | I |
|  | 3 | S | R | R | S | R | S | S | S |
|  | 4 | S | R | R | S | R | S | S | S |
| South-West Pacific | 1 | S | S | S | S | S | S | S | S |
|  | 2 | S | S | S | S | S | S | S | S |
|  | 3 | S | S | S | S | S | S | S | S |
|  | 4 | S | S | S | S | S | S | S | S |
|  | 5 | R | S | S | S | S | S | S | I |
| USA600 | 1 | R | R | R | S | R | S | S | S |
|  | 2 | R | R | R | S | R | S | S | S |
|  | 3 | R | R | R | S | R | S | S | S |
| Berlin | 1 | S | R | S | S | R | S | S | I |
|  | 2 | S | S | S | S | R | S | S | S |
|  | 3 | S | S | S | S | R | S | S | I |
|  | 4 | S | S | S | S | R | S | S | S |
|  | 5 | S | S | S | S | R | S | S | S |
|  | 6 | S | R | R | S | R | S | S | S |
|  | 7 | S | R | R | S | R | S | S | I |
|  | 8 | S | S | S | S | R | S | S | I |
|  | 9 | S | S | S | S | R | S | S | S |
|  | 10 | S | S | S | S | S | S | S | I |
|  | 11 | S | R | R | S | R | S | S | S |
| Southern Germany | 1 | S | R | R | S | R | S | R | R |
|  | 2 | S | R | S | S | S | S | S | S |
|  | 3 | S | R | R | S | R | S | S | R |
|  | 4 | S | R | R | S | R | S | S | R |
|  | 5 | S | R | R | S | S | S | S | R |
|  | 6 | S | R | R | S | R | S | S | R |
| New York/Japan | 1 | S | R | R | S | R | S | S | S |
|  | 2 | S | R | R | S | R | R | S | S |
|  | 3 | S | R | R | S | R | R | S | S |
|  | 4 | S | R | R | S | R | R | S | I |
| pediatric | 1 | S | S | S | S | S | S | S | S |
|  | 2 | S | S | S | S | S | R | S | S |
|  | 3 | S | S | S | S | S | S | S | I |
|  | 4 | S | S | S | S | R | S | S | I |
|  | 5 | R | R | S | S | R | R | S | S |
| European | 1 | S | S | S | S | S | S | S | S |
|  | 2 | S | S | S | S | S | S | S | I |
|  | 3 | R | S | S | S | S | S | S | S |
|  | 4 | S | S | S | S | S | S | S | I |
| Iberian | 1 | S | R | R | S | R | R | R | R |
|  | 2 | S | R | R | S | R | S | S | I |
|  | 3 | S | R | R | S | R | R | R | R |
|  | 4 | S | R | R | S | R | R | R | R |
| Hungarian/Brazilian | 1 | S | R | R | S | R | R | S | R |
|  | 2 | S | R | S | S | S | R | S | R |
|  | 3 | S | R | R | S | S | R | S | R |
|  | 4 | R | R | S | R | R | R | S | R |
|  | 5 | S | R | S | S | R | R | S | R |
|  | 6 | S | R | R | S | R | R | S | R |
|  | 7 | S | R | R | R | R | R | S | R |
|  | 8 | S | R | R | S | S | R | S | R |
|  | 9 | S | R | R | S | S | R | S | R |
|  | 10 | R | R | R | R | R | R | S | R |
|  | 11 | S | R | R | S | S | R | S | R |
|  | 12 | S | R | R | R | R | R | R | R |
| USA500 | 1 | S | R | R | S | R | S | S | I |
|  | 2 | S | R | R | S | R | S | S | S |
|  | 3 | S | R | R | S | R | S | S | S |
|  | 4 | S | R | R | S | R | S | S | S |
|  | 5 | S | S | S | S | R | S | S | S |
|  | 6 | S | S | S | S | R | S | S | S |
|  | 7 | S | R | S | S | R | S | S | I |
|  | 8 | S | R | R | S | R | S | S | S |
| USA300 | 1 | S | R | S | S | S | R | S | S |
|  | 2 | S | R | S | S | R | S | S | S |
|  | 3 | S | R | S | S | S | S | S | S |

**Table S1**: Antibiotic susceptibility data of MRSA clones
